# Therapist Coaching in Parent-Child Interaction Therapy in the Netherlands: An Archival Lag Sequential Analysis Study

**DOI:** 10.1177/01454455251319731

**Published:** 2025-03-12

**Authors:** Iza C. A. Scherpbier, Mariëlle E. Abrahamse, Mirte N. Mos, Ramón J. L. Lindauer, Larissa N. Niec

**Affiliations:** 1 Amsterdam UMC Locatie AMC, The Netherlands; 2 Karakter, Ede, Gelderland, The Netherlands; 3 Central Michigan University, Mount Pleasant, USA

**Keywords:** parent-child interaction therapy, lag sequential analysis, therapist coaching, parenting skills, therapist-parent dyad

## Abstract

In vivo therapeutic coaching of parent-child interactions is the primary mechanism of change in behavioral parent training programs such as parent-child interaction therapy (PCIT), yet relatively little research has examined the coaching process. The primary aim of this study was to explore the bidirectional interaction between therapist-parent dyads to better understand how therapists influence parent behavior and vice versa. Observational data from two research projects were analyzed separately and together using lag sequential analysis (LSA). Results demonstrate that therapist responsive coaching (e.g., praising parent behavior) led parents to use more child-centered skills. Responsive coaching techniques led to immediate increases in parents’ use of the targeted positive parenting skill (10%–25% re-use). Responsive strategies followed targeted parent verbalizations more often than directive strategies, suggesting that therapists reinforce positive parenting skills as soon as parents use them. When directive coaching techniques were used, there was a 18% to 32% chance that parents followed through with a child-centered skill as coached. This study is the first to explore the influence of in vivo coaching on parent skill acquisition on a micro-level and has implications for the therapist training.

## Introduction

In vivo therapeutic coaching of parent–child interaction is a powerful mechanism of change that builds parenting skills in behavioral parent training (BPT) programs such as parent-child interaction therapy (PCIT; Barnett et al., 2014; Heymann et al., 2022). Specifically, therapists use behavioral principles to shape parents’ skills during play interactions with their children. Through live coaching that can be provided during sessions in clinic, at home, or via telehealth, therapists reinforce parents’ child-centered skills and decrease leading behaviors (Barnett et al., 2017). Although therapeutic in vivo coaching is a core component of PCIT, few studies have examined the coaching process (Barnett et al., 2014, 2017; Heymann et al., 2022). Existing investigations indicate that in vivo coaching significantly and meaningfully increases parenting skills (Heymann et al., 2022; Shanley & Niec, 2010). However, to our knowledge, no studies have investigated the bidirectional interaction between therapist coaching and parent behavior.

Therapeutic coaching strategies can be grouped into two primary categories: directive and responsive. Directive coaching comes before a parent verbalization, telling a parent what to do or say (e.g., “Go ahead and praise him for staying calm.”). Directive coaching techniques, such as modelling or prompting a positive parenting skill, are considered particularly useful early in treatment in order to stimulate parents to use positive parenting skills when they do not yet know how to use them spontaneously (Barnett et al., 2018). Responsive coaching comes after a parent verbalization and typically reinforces the parent’s use of a skill (e.g., “Great reflection!”; Barnett et al., 2014). Responsive coaching mediates the acquisition of positive parenting skills from one session to the next and predicts treatment completion (Barnett et al., 2014, 2017; Green Rosas et al., 2022). In contrast, directive coaching has been related to treatment attrition (Heymann et al., 2022) and poor acquisition of the child-centered skills (Barnett et al., 2014). Results from multiple studies therefore conclude and encourage therapists to use responsive techniques to reinforce parent skill use while minimalizing directive techniques early on. Nonetheless, we cannot forget that while there is a focus on the interaction between therapists and parents within the coaching context, the Dyadic Parent-Child Interaction Coding System (DPICS) coding that is conducted at the beginning of each session (i.e., a five-minute observation period where parent and child verbalizations are tallied) inherently impacts coaching behavior for the remainder of the session. That is, if a parent uses fewer positive parenting skills during the initial observation period, it is likely that during actual coaching, there will be fewer verbalizations of positive parenting skills for a therapist to respond to. This may result in therapists initiating some directive coaching statements to promote parent verbalizations that they can subsequently reinforce via responsive coaching techniques.

These correlational studies give us a first insight into the associations between coaching strategies and parental skill acquisition. However, as coaching is considered to be an active mechanism of change in PCIT, it is important to delve deeper into the dynamic interaction process between therapist and parent. In order to do so, the current study used lag sequential analysis (LSA) to explore the bidirectional influence of therapist coaching and parent verbalizations. Using this technique allows for the discovery of patterns in therapist and parent verbalizations. Understanding the interaction between therapist and parental verbalizations can provide insight into the ways in which therapists influence parent behavior and parents influence therapist behavior. This has implications for therapist training and could potentially improve implementation of BPT programs such as PCIT.

The primary aim of the current study was to explore the bidirectional interaction between therapist and parent verbalizations during the five-minute coaching period in the first three Child-Directed Interaction (CDI) sessions of PCIT (i.e., first phase of PCIT). The first three sessions were chosen to examine as previous studies of therapist coaching have found the early sessions in the child-directed phase of PCIT to relate to important family outcomes (Barnett et al., 2014; Hakman et al., 2009; Shanley & Niec, 2010). Using LSA, we examined whether responsive and directive coaching sequentially predicted what parent verbalizations preceded and succeeded therapists’ coaching by PCIT therapists in the Netherlands. We expected that responsive coaching strategies were used more often than directive strategies. Moreover, we expected that responsive coaching techniques more often succeeded parent verbalizations than directive coaching techniques. Finally, we expected that responsive coaching techniques led to more positive parenting skills than directive coaching techniques.

## Methods

### Procedure

The current study included the analysis of archival data from two previous studies of the efficacy of PCIT in the Netherlands (Abrahamse et al., 2016, 2021). Therapist-parent interactions and parent–child interactions were coded during the first three CDI coaching sessions of 17 families using the Therapist-Parent Interaction Coding System (TPICS) and the Dyadic Parent-Child Interaction Coding System (DPICS; Eyberg et al., 2004; Niec et al., 2016). As mentioned, the first three sessions were chosen to examine as previous studies of therapist coaching have found the early sessions in the child-directed phase of PCIT to relate to important family outcomes (Barnett et al., 2014; Hakman et al., 2009; Shanley & Niec, 2010).

The coach coding began two minutes after the therapist started coaching the caregiver and stopped when five minutes of coaching time was recorded. All coaching verbalizations and parental verbalizations were coded. If the coaching was interrupted (e.g., due to a bathroom break), the coding was continued after the disruption until there was five minutes of coaching coded. All verbalizations were scored using Noldus Observer (Noldus, 1991), which is a software system for collection and analysis of observational data. Results were analyzed using LSA in Excel and IBM SPSS Statistics (Meyers et al., 2013).

### Treatment

As an archival study, the participants were gathered from two separate research projects (Abrahamse et al., 2016, 2021), where the treatment varied in its delivery format: standard in clinic PCIT and PCIT-Home. These two formats are described below. Importantly, the first three sessions of treatment which are included in the study do not vary across formats. Thus, the setting (in clinic or in home) was the primary factor that varied across the two datasets.

#### Standard PCIT

PCIT includes two phases: the first phase (Child-Directed Interaction [CDI]) focuses on enhancing parents’ child-centered interaction skills, and the second phase (Parent-Directed Interaction [PDI]) focuses on helping parents to set safe and effective limits for their children. During CDI, parents are taught to increase positive parenting skills, such as praises, reflections and behavior descriptions, while decreasing parents’ leading verbalizations such as questions, commands and criticisms. Parents complete the CDI phase when skill acquisition is achieved during a weekly five-minute standardized observation time. During the PDI phase of treatment, parents learn how to give effective commands and implement developmentally appropriate limit-setting strategies such as time-out from positive reinforcement (Eyberg & Funderburk, 2011). PCIT does not have a set number of sessions, but rather moves forward as parents demonstrate sufficient acquisition of skills.

#### PCIT-Home

PCIT-Home includes all the same components as standard PCIT. Two primary differences exist: first, the treatment is provided to families in their home, meaning that the therapist coaches the parent at home from a small distance, rather than through a one-way mirror and through a microphone and earpiece. Second, the number of sessions in PCIT-Home is limited to eight, with the first four sessions devoted to the CDI phase, and the last four sessions devoted to the PDI phase. For the first three sessions, therapists providing in-home PCIT used the same protocol as the therapists providing standard PCIT. Moreover, therapists are not attempting to get parents to goal skill criteria within four CDI or PDI sessions.

### Participants

#### Families

The records of seventeen families were selected for the study based on the availability of the video-recorded therapy sessions. Seven families were selected from the research project offering standard PCIT and ten families were selected from the research project offering PCIT-Home (see Table 1).

**Table 1.** Descriptive Statistics of Children and Parents in Standard PCIT and PCIT-Home Samples.

Descriptives	Standard PCIT		PCIT-home		Total sample	
	*n*		*n*		*n*	
Children						
Age in years (*M*, *SD*)	7	5.64, 1.54	10	5.96, 1.42	17	5.83, 1.43
Sex (% male)	7	71	10	70	17	71
Country born (% the Netherlands)	7	100	10	80	17	88
Ethnicity (% Western)	7	57	10	50	17	53
ECBI_Mothers_ Intensity scale baseline (*M*, *SD*)	7	146.57, 25.54	9	131.33, 29,28	16	138.00, 33.83
ECBI_Mothers_ Problem scale baseline (*M*, *SD*)	7	14.00, 9.88	9	19.67, 8.53	16	17.19, 9.29
Parents						
Included parent (% mothers)	7	57	10	100	17	82
Family status (% single-parent)	7	43	10	70^a^	17	59
Education level mother (% college or university)	6	50	8	38	14	43

*Note.* The difference in the ECBI scales between the two samples are not significant (*p*_Intensity_ = .390, *p*_Problem_ = .239).

a There is one participant with missing information for family status.

In the PCIT-Home sample, there were two families where only two coaching sessions were available to code, meaning that a total of 49 videos were coded during five-minutes of coaching time. For each child, one corresponding parent used for the analyses. Whether the mother or father was used for this archival study, was purely based on availability and quality of the video-recording.

#### Therapists

Eleven therapists coached the families. Six therapists treated one family each, four therapists treated two families each, and one therapist treated three families. The years of therapist experience in PCIT varied between 2 and 12 years (*M* = 7.7). All PCIT therapists were trained and certified by a PCIT International-certified trainer. The training process included an initial 40-hour workshop and subsequent mandatory bi-weekly consultation with a PCIT Global Trainer, allowing therapists to give PCIT in the Netherlands. All therapists completed their training between 2008 and 2013, which was prior to the availability of PCIT International therapist certification.

### Measures

#### Dyadic Parent-Child Interaction Coding System-III

The DPICS-III (Abrahamse et al., 2019; Eyberg et al., 2004) is a well-validated behavioral observation coding system that evaluates the quality of the parent-child interactions and includes child-centered (e.g., praises, reflections) and leading (e.g., questions, criticisms) parent verbalizations. For the purposes of this study, we wished to examine the sequences of therapist and parent verbalizations, thus we coded the parents’ verbalizations during the first five minutes of coaching. The following DPICS categories were used: Behavior Description (BD), Labeled Praise (LP), Unlabeled Praise (UP), Reflection (RF), Question (Qu), Negative Talk (NTA), Indirect Command (IC), and Direct Command (DC). For data reduction, we used two DPICS composites: (1) “Positive Following,” which consists of BD, LP, UP and RF, and “Negative Leading,” which is made up of Qu, NTA, IC, and DC. We assessed the parents’ verbalizations in CDI session 1, 2 and 3. The DPICS manual was translated to Dutch (Abrahamse et al., 2019) and the videos were coded in Dutch.

#### Therapist-Parent Interaction Coding System

The TPICS (Niec et al., 2016) is a behavioral observation coding system designed to assess therapists’ coaching strategies during PCIT. Coaching techniques coded by the TPICS can be classified as directive or responsive. An example of a directive coaching technique is modeling, where the therapist verbalizes a PCIT parenting skill as an example (e.g., “Thank you for sharing with me.”). Further directive coaching techniques are: direct and indirect commands, child observations, drills, prompting, and rationale remarks (see Table 2). An example of a responsive coaching technique is a labeled praise, where a therapist positively evaluates a specific behavior, verbalization or activity of the parent (e.g. “That was great behavior description you gave him.”). Further responsive coaching techniques are: unlabeled praises, process comments, reflective descriptions, corrective criticism, assurance comments, and exclusion explanations (see Table 2). The TPICS predicts parents’ skill acquisition from one session the next as well as speed of completion of the first phase of treatment. TPICS reliability has been found to be excellent (Barnett et al., 2014). The TPICS manual was translated to Dutch and the videos were coded in Dutch.

**Table 2.** Directive and Responsive Coaching Strategies, Their Definition and an Example of Strategy Application.

Coaching strategies	Definition	Example
Directive strategies		
Modeling	The therapist verbalizes an example of a parenting skill	“Thank you for picking up your toys”
Direct command	The therapist declaratively states a direction for the parent to perform a behavior	“Praise her for picking up her toys”
Indirect command	The therapist suggests a direction for the parent to perform a behavior	“Let’s try a behavior description”
Child observation	The therapist makes any observation about the child to draw the parent’s attention to their child	“She just came back to the table”
Drill	An exercise during which the therapist tells the parent to use a parenting skill for a specific duration and/or frequency	“We are going to focus on behavior descriptions, give as many as you can in the next 2 minutes”
Prompting	The therapist verbalizes the beginning of a skill with the intention of letting the parent finish the statement	“Thank you for. . .”
Rationale remark	The therapist makes a statement to teach the parent about PCIT or parenting skills, their goal or about (not yet) exhibited parent behavior	“By staying calm yourself, you are setting a good example for her”
Responsive strategies		
Labeled praise	The therapist verbalizes a specific positive evaluation of a skill use, behavior or activity by the parent	“Beautiful behavior description!”
Unlabeled praise	The therapist verbalizes a generic positive evaluation of the parent or the parent’s behavior	“Well done”
Process comment	The therapist connects the child’s behavior to the parent’s treatment-related behavior through a statement	“She smiled when you praised her”
Reflective description	The therapist verbalizes a non-evaluative statement about the parent’s most recent skill use or behavior	“That was a behavior description”
Corrective criticism	The therapist is gently critical of the parent’s behavior	“Oops, that was a question”
Assurance comment	The therapist addresses a parent or child behavior to normalize or reframe the parent’s thought or feeling	“I know that it is difficult to stay calm while she is yelling”
Exclusion explanation	The therapist makes a statement about a skill or behavior that should be avoided. The statement is connecting a parent’s past/present/future behavior to their child’s behavior, the situation or the treatment goals	“Avoid criticism, as this can damage her self-esteem”

#### Noldus Observer

All therapist and parent verbalizations, as dictated by the TPICS and DPICS, were coded using Noldus Observer (Noldus, 1991). This is an observational coding program used to code behaviors in a sequence. Noldus Observer is a very precise tool that codes on the second. Hence, researchers decided to use a tolerance rate of five seconds in coding. A second coder coded 25% of the videos, after which an interrater reliability analysis was run. This resulted in *k* = .55 with a *k_max_* = .82. In this sample, we used a *k_max_* to contextualize the value of *k* and to indicate the agreement between coders within the marginal distribution of the sample This resulted in an interrater reliability of approximately 67% of the maximum possible agreement, indicating a substantial agreement.

#### Eyberg Child Behavior Inventory

The ECBI (Eyberg & Pincus, 1999) is a parent-rating scale of child conduct problems. It contains 36 items and was designed to assess the disruptive behaviors of children between the ages of 2 and 16 years. Two scales are included in the measure: the Intensity Scale; a 7-point scale from 1 (never) to 7 (always) and Problem Scale; a dichotomous scale with 1 (yes) and 0 (no). The Dutch-language ECBI has good psychometric qualities (Abrahamse et al., 2015). We used the ECBI to check whether the levels of children’s disruptive behavioral problems were significantly different from one another at baseline, in order to see whether we could assume similarities between the two research samples at baseline. We found that the difference in Intensity and Problem Scales were not significant at baseline (*p_Intensity Scale_* = .286 and *p_Problem Scale_* = .250).

### Data Analysis Plan

IBM SPSS Statistics 29 (Meyers et al., 2013) and Noldus Observer (Noldus, 1991) were used for the analyses. Because of slight differences in the implementation of PCIT across the two samples, the analyses in SPSS were conducted separately for each sample as well as for the combined sample. The analyses in Noldus Observer were only conducted on the combined sample. First, a General Linear Model (GLM) was used to examine the development of parenting skills across the first three CDI sessions to determine whether there were differences across the two PCIT research samples. The dependent variable was parenting skills (Positive Following or Negative Leading), and the independent variable was CDI session (session 1, 2 or 3). Prior to conducting the GLM, assumptions were checked and there were no significant violations. Secondly, pairwise *t*-tests were conducted to compare coaching strategies (responsive and directive) between CDI sessions. Assumptions were checked and there were no significant violations. This meant that the analysis proceeded with parametric tests. Thirdly, LSA was used to determine sequences in parent and therapist verbalizations. In order to identify what respective verbalization immediately preceded or followed one other, LSA plus and minus one was used.

## Results

### Changes in Parenting Skills

#### How Do Parenting Skills Develop Over the First Three Treatment Sessions?

Frequencies of parents’ Positive Following and Negative Leading were calculated for each sample. Parent verbalizations were coded during from the same five-minute coding period used to assess therapist coaching (see Table 3).

**Table 3.** Participants, Means, and Standard Deviations of DPICS Positive Following and Negative Leading Measured With DPICS Parenting Skills Over Sessions 1, 2 and 3.

Measure	Standard PCIT			PCIT-home			Total sample		
	*n*	*M*	*SD*	*n*	*M*	*SD*	*n*	*M*	*SD*
DPICS positive following									
Session 1	7	19.86	9.42	8	29.25	12.60	15	24.87	11.87
Session 2	7	19.00	12.44	8	22.63	6.70	15	20.93	9.60
Session 3	7	18.43	9.05	8	26.88	13.28	15	22.93	11.93
DPICS negative leading									
Session 1	7	16.43	13.05	8	8.25	5.55	15	12.07	10.31
Session 2	7	6.43	4.61	8	8.25	5.65	15	7.40	5.10
Session 3	7	4.86	3.53	8	4.63	4.87	15	4.73	4.15

*Note*. There was one outlier in the DPICS Positive Following session 3 Standard PCIT sample. There are two outliers in the DPICS Negative Leading session 3, one in each sample.

Among families who received PCIT in the clinic, there was no significant difference in DPICS Positive Following between sessions 1, 2 and 3 (*F*(2, 12) = .05, *p* = .956, partial *η*^2^ = .007). A significant effect was found in DPICS Negative Leading between sessions 1, 2 and 3 (*F*(2, 12) = 7.10, *p* = .009, partial *η*^2^ = .542). Similarly, for families who received PCIT in the home, there was also no significant difference in DPICS Positive Following from sessions 1, 2 and 3 (*F*(2, 14) = 1.73, *p* = .213, partial *η*^2^ = .198). There was a non-significant difference in DPICS Negative Leading between sessions 1, 2 and 3 (*F*(2, 14) = 2.09, *p* = .161, partial *η*^2^ = .230). Although the DPICS Negative Leading patterns were different between the in clinic and home samples, we believe that due to the similar mean in session three (*M_s3 in clinic_* = 4.86, and *M_s3 in home_* = 4.63), this difference is acceptable. In other words, treatment patterns looked similar across the two treatment settings.

### Therapist Coaching

#### What Coaching Strategies Do PCIT Therapists Use During CDI?

In order to determine whether there was a difference between therapist verbalizations over the first three CDI sessions in the two samples from the different research projects, the frequencies of therapist responsive and directive coaching, and neutral talk were first reported separately. The total amount of verbalizations in the two samples together was subsequently reported and analyzed together, as there was a small difference between the use of coaching in each sample.

##### Standard PCIT Sample

In the first CDI coaching session, a mean of 51% of total therapist verbalizations were responsive, 31% were directive and 18% were neutral talk. In the second session, therapists on average, made 45% responsive verbalizations, 30% directive verbalizations and 25% neutral talk verbalizations. In the third session, therapists had an average of 51% responsive verbalizations, 31% of directive verbalizations and 18% neutral talk verbalizations.

There were no significant in-group differences between the therapists’ use of responsive techniques in session 1 (*M* = 20.00, *SD* = 11.31) compared to session 3 (*M* = 21.43, *SD* = 14.12); (*t*[6] = −.266, *p* = .400, *d* = −.101), with a non-significant positive relationship; (*r*[7] = .392, *p* = .192). Results also showed that there was no significant in-group difference between the therapists’ use of directive techniques in session 1 (*M* = 10.71, *SD* = 8.50) compared to session 3 (*M* = 11.86, *SD* = 5.21); (*t*[6] = −.395, *p* = .353, *d* = −.149). There was also a non-significant positive relationship; (*r*[7] = .462, *p* = .148).

##### PCIT-Home Sample

In the first CDI coaching session, a mean of 53% of total therapist verbalizations were responsive, 27% were directive and 20% were neutral talk. In the second session, therapists on average, made 54% responsive verbalizations, 23% directive verbalizations and 23% neutral talk verbalizations. In the third session, therapists had an average of 45% responsive verbalizations, 29% of directive verbalizations, and 26% neutral talk verbalizations.

There was no significant in-group difference between the therapists’ use of responsive techniques in session 1 (*M* = 24.00, *SD* = 5.45) compared to session 3 (*M* = 18.63, *SD* = 9.84); (*t*[7] = 1.505, *p* = .088, *d* = .531), with a non-significant positive relationship; (*r*[8] = .229, *p* = .293). Results also showed that there was no significant in-group difference between the therapists’ use of directive techniques in session 1 (*M* = 11.25, *SD* = 8.26) compared to session 3 (*M* = 10.75, *SD* = 9.18); (*t*[7] = .258, *p* = .402, *d* = .091), but that there was a significant positive relationship; (*r*[8] = .808, *p* = .008).

##### Total

In the first CDI coaching session, a mean of 52% of total therapist verbalizations were responsive, 29% were directive and 19% were neutral talk. In the second session, therapists on average, made 50% responsive verbalizations, 26% directive verbalizations and 24% neutral talk verbalizations. In the third session, therapists had an average of 47% responsive verbalizations, 30% of directive verbalizations, and 23% neutral talk verbalizations.

Overall, there were no significant differences between the therapists’ use of responsive techniques in session 1 (*M* = 22.13, *SD* = 8.60) compared to session 3 (*M* = 19.93, *SD* = 11.66); (*t*[14] = .696, *p* = .249, *d* = .180), with a non-significant positive relationship; (*r*[15] = .299, *p* = .139). Results also showed that there was no significant difference between the therapists’ use of directive techniques in session 1 (*M* = 11, *SD* = 8.07) compared to session 3 (*M* = 11.27, *SD* = 7.35); (*t*[14] = −.162, *p* = .437, *d* = −.042), but that there was a significant positive relationship; (*r*[15] = .661, *p* = .004).

Behavior analysis were subsequently performed through Noldus Observer (Noldus, 1991), which allowed us to split the responsive and directive coaching strategies into the subcategories of techniques per strategy, and demonstrated how often therapists used those. In absolute numbers, from the responsive techniques, therapists most often use labeled praises (573 times), unlabeled praises (267 times), process comments (80 times) and reflective descriptions (63 times). All other responsive techniques (corrective criticisms, assurance comments, and exclusion explanations) were also used, but all under 50 times in total over all sessions. From the directive techniques, therapists most often used modeling (242 times), child observations (100 times), indirect commands (100 times), and direct commands (89 times). All other techniques (drills, prompting, and rationale remarks) were also used, but all under 50 times in total over all sessions.

#### What Is the Likelihood That a Coaching Technique Precedes Or Succeeds a Parent’s Verbalization?

##### Lag Sequential Analysis

Using LSA in Noldus Observer (Noldus, 1991), we evaluated what the probability was that a therapist coaching technique would either precede or succeed a parent’s verbalization and more specifically, which techniques and verbalizations. Due to the facts that separating the two samples (Standard PCIT and PCIT-Home) would have caused the study to be underpowered, and that there was little difference between the use of therapist verbalizations between the two samples, the entire sample is analyzed as a whole in the LSA.

**Figure 1.**
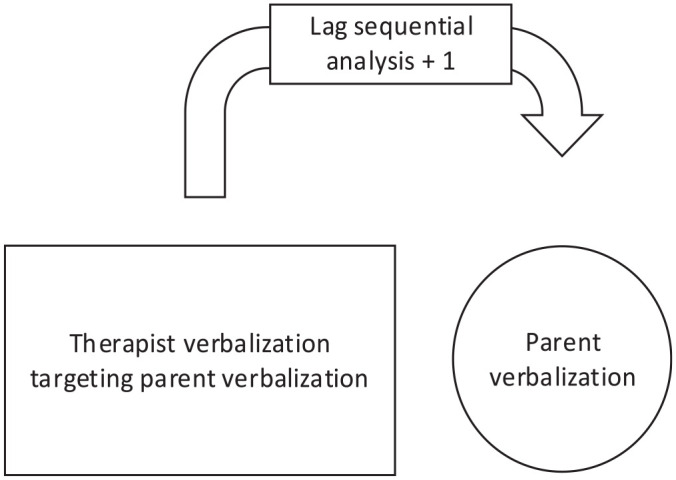
Lag sequential analysis plus one: The chance that the respective therapist verbalization targeting a specific parent verbalization preceded the respective targeted parent verbalization.

**Figure 2.**
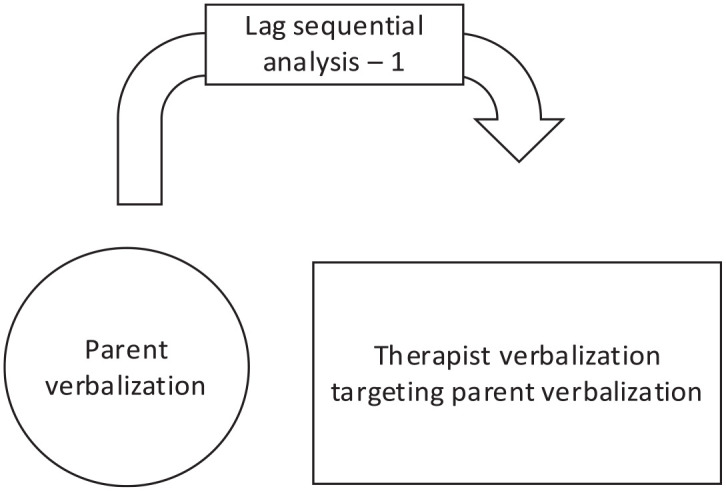
Lag sequential analysis minus one: The chance that the respective therapist verbalization targeting a specific parent verbalization followed the respective targeted parent verbalization.

The four responsive and four directive techniques that were most often used by therapists were analyzed. Of the responsive coaching strategy, the most used techniques were labeled praises (LP), reflective descriptions (RF), unlabeled praises (UP), and process comments (PC). Of the directive coaching strategy, the most used techniques were modeling (Mo), child observations (CO), indirect commands (IC), and direct commands (DC). Figures 1 and 2, and Tables 4 and 5 indicate what the probability (expressed in percentage) was that a respective therapist coaching technique was given before a targeted parenting skill (LSA plus one), and what the probability was that a respective therapist coaching technique was given after a targeted parenting skill (LSA minus one).

**Table 4.** The Chance (%) That the Respective Therapist Verbalization Targeting a Parent Verbalization Preceded the Respective Targeted Parent Verbalization.

Therapist coaching techniques	Targeting BD	Targeting RF	Targeting LP	Targeting UP	Targeting Qu
Responsive techniques					
Therapist LP	21.4	15.8	10.9	17.9	—^a^
Therapist RD	18.5	25.0	12.5	0.0	16.7
Therapist UP	0.0	10.4	4.5	5.8	4.5
Therapist PC	0.0	0.0	0.0	0.0	0.0
Directive techniques					
Therapist Mo	32.3	29.8	31.3	20.0	—^a^
Therapist DC	29.4	30.0	28.6	—^a^	—^a^
Therapist IC	22.2	0.0	18.2	—^a^	0.0
Therapist CO	5.4	9.0	5.4	3.6	4.5

*Note.* LP = labeled praise; RD = reflective description; UP = unlabeled praise; PC = process comment; Mo = modeling; DC = direct command; IC = indirect command; CO = child observation; BD = behavior description; RF = reflection; Qu = question.

a Did not happen in sample.

**Table 5.** The Chance (%) That the Respective Therapist Verbalization Targeting a Parent Verbalization Followed the Respective Targeted Parent Verbalization.

Therapist coaching techniques	Targeting BD	Targeting RF	Targeting LP	Targeting UP	Targeting Qu
Responsive techniques					
Therapist LP	60.9	72.5	60.7	67.7	—^a^
Therapist RD	44.0	46.7	42.9	66.7	56.3
Therapist UP	14.5	12.3	11.4	4.6	2.5
Therapist PC	0.0	4.3	1.4	50.0	0.0
Directive techniques					
Therapist Mo	7.7	14.6	2.3	0.0	—^a^
Therapist DC	2.9	10.0	0.0	—^a^	—^a^
Therapist IC	0.0	7.1	0.0	—^a^	0.0
Therapist CO	1.8	6.7	2.5	5.0	4.2

*Note*. LP = labeled praise; RD = reflective description; UP = unlabeled praise; PC = process comment; Mo = modeling; DC = direct command; IC = indirect command; CO = child observation; BD = behavior description; RF = reflection; Qu = question.

a Did not happen in sample.

Example of how to read the table: Within all therapist LP-verbalizations that targeted a parent BD, there were 21.4% of those verbalizations that preceded a parent BD. The remaining 78.6% of the therapist LP-verbalizations that targeted a parent BD, preceded other parent and therapist verbalizations. This means that when the table says “Therapist LP” and the column that says “Targeting BD,” it is only coding when a therapist used an LP for a parent BD, which was followed by another parent BD.

Example of how to read this table: Within all therapist LP-verbalizations that targeted BD, there were 60.9% of those verbalizations that followed a parent BD. This means that the remaining 39.1% of therapist LP-verbalizations that targeted BD followed other parent and therapist verbalizations. This means that when the table says “Therapist LP” and the column says “Targeting BD,” it is only coding when the therapist actually used an LP for a parent BD, after a parent had used a BD.

## Discussion

The current study aimed to explore the bidirectional interaction between therapist and parent verbalizations in PCIT. More specifically, the study sought to investigate which parent verbalizations preceded and succeeded therapists’ coaching through lag sequential analysis. To do so, we first identified which coaching strategy was most used in the first three CDI sessions and which techniques within the responsive and directive coaching strategies were most frequently used by therapists. Data was gathered from two research projects and the samples were therefore analyzed both separately and as a combined sample. Findings suggest that in the first three CDI sessions, the responsive coaching strategy was used more by therapists than the directive coaching strategy. There were no significant in-group differences (in either research sample) in therapists’ use of responsive nor directive techniques across sessions. We did however, find a positive relationship in the use of directive techniques from sessions 1 to 3. In other words, if more directive techniques were used in session 1, it tended to also be used more in session 3. Regarding parenting skills, positive following did not significantly change across sessions, but negative leading decreased over time. The findings from the sequential analyses can be split up into looking at preceding and succeeding verbalizations lag sequentially for both responsive and directive coaching techniques. For the responsive therapist coaching techniques preceding targeted parent verbalizations, we found that when labeled praises and reflective descriptions preceded a targeted parent verbalization, the probability that a targeted skill was subsequently re-used, was between 10% and 25%. This means that these responsive coaching techniques led to immediate increases in parents’ use of the targeted positive parenting skill. For the directive therapist coaching preceding targeted parent verbalizations, we found that when modeling, indirect and direct commands preceded a targeted parent verbalization, the probability that a targeted parent verbalization was subsequently re-used, was between 18% and 32%. This means that parents were quite likely to follow through with a positive parenting skill requested by a therapist. The findings from lag sequentially succeeding verbalizations illustrate that responsive therapist verbalizations follow targeted parent verbalizations far more often than directive therapist verbalizations. This suggests that therapists immediately reinforce parents in their use of positive parenting skills.

Previous correlational studies have begun to address the association between coaching strategies and parental skill acquisition (Barnett et al., 2014, 2017, 2018). The current study sought to build on these first investigations by looking at the dynamic interaction process between therapists and parents through lag sequential analysis. With that, this study is the first to look at coaching in PCIT at a micro and bidirectional level. Lag sequential analysis gave us insight into what preceded and succeeded a parent verbalization. We did not find support for our hypothesis that responsive coaching techniques would lead to more positive parenting skills than directive coaching techniques. Perhaps this is because directive techniques are in fact designed to evoke wanted behavior, for example through modeling what the therapist wishes for the parent to say. In our study, when therapists used modeling, direct and indirect commands that targeted parent’s behavior descriptions, reflections and labeled praises, these parenting skills were evoked. To be specific, parents used child-centered skills after directive coaching two to three times more than after a responsive coaching strategy. This means that in these first CDI sessions, parents benefit from receiving concise suggestions to produce wanted skills. Therefore, it was unsurprising to find that directive techniques preceded parenting skills more often than responsive techniques. Moreover, we were intrigued to find that the chance a responsive technique preceded a targeted child-centered skill, was also quite high. As seen in the lag sequentially preceding verbalizations, responsive coaching strategies had an immediate effect on the first following parent verbalization. As such, we found that when a therapist gave a labeled praise for a parent’s use of a child-centered skill, parents were likely to follow that up with the same skill immediately after. This suggests that responsive coaching reinforces parents to re-use positive parenting skills that are being coached. Furthermore, this finding confirms previous research suggesting that a higher rate of responsive coaching predicted faster skill acquisition (Barnett et al., 2017; Heymann et al., 2022).

As expected, responsive coaching, and hence responsive coaching techniques were most frequently employed in the first three sessions. The most used responsive techniques were labeled and unlabeled praises, process comments and reflective descriptions, which are all techniques that commend and encourage a parent in their skill use. More specifically, previous research showed that praising parents not only predicted an increased acquisition rate of positive parenting skills, but also that positive feedback is a vital part of acquiring new skills as it stimulates further skill use (Eyberg & Funderburk, 2011; Heymann et al., 2022). Additionally, in PCIT, therapists can actively provide positive social reinforcement to target wanted behavior changes (Greco et al., 2001). So, when a therapist praises a parent for using a positive parenting skill, it reinforces its use. As such, a therapist “models” how to praise by praising a parent for their behavior, which consequently leads to further parental application of praises. In other words, our study shows that there is a parallel process of positive encouragement happening in therapist-parent dyads and parent-child dyads, which is also encouraged through the way the PCIT protocol is set up (Eyberg & Funderburk, 2011). This finding is clinically relevant, as positively modeling behavior can reinforce desired outcomes and boost parents’ confidence and motivation. It was previously found that greater therapist-parent alliances led to greater improvements in parenting practices at the end of parenting interventions, and that therapists’ use of skills led to a decrease in parenting stress (Leitão et al., 2021). With that, we can carefully imply that when parents feel heard and supported by the therapist, they may be more likely to replicate this positive encouragement to their children. On top of that, multiple meta-analyses have shown that positive reinforcement, and specifically praising, helps reduce child disruptive more effectively than when this is lacking (Kaminski et al., 2008; Leijten et al., 2019). We believe that this parallel process of positive encouragement should be addressed during therapist training to help therapists understand the impact their encouragement can have in the therapeutic alliance, the treatment progress, and that it can ultimately lead to better outcomes in the parent-child interactions.

When specifically zooming in on reflective descriptions, it was interesting to find that the chance that a reflective description was given after a parent verbalized a question, was above 50%. This alerts parents to their use of leading verbalizations through a mere statement—“that was a question,” rather than through a correction or criticism. This is a positive way to approach and encourage behavior change. Furthermore, visible in lag sequential preceding verbalizations, the chance that a directive technique follows a positive parenting skill is less than 15% for any respective skill in this sample, which suggests that therapists tended to stay away from giving direction for the next verbalization soon after child-centered skill was used. It would be interesting to conduct future research with expanded sequences such as lag sequential plus two, to discover whether a therapist uses a directive coaching technique secondarily, or whether a different pattern emerges here.

Although this study is the first to use lag sequential analysis in PCIT and helps us to understand what therapist verbalizations activate skill acquisition, it is important to address some limitations. Firstly, the fact that the samples came from two different research projects (although both clinical samples) where the intervention setting differed, must be acknowledged. For transparency, we evaluated the ECBI baseline scores, DPICS scores and frequency of coaching strategies both separately for the research projects and as a combined sample. Although there were some slight differences in the ECBI and DPICS scores, the decision was made to bundle the participants for the lag sequential analysis nonetheless, as separating the research projects would have caused too low a power to analyze. However, this limitation does mean that the results of the lag sequential analysis must be interpreted with caution, and warrants replication (Abrahamse et al., 2019). Furthermore, the study had a small sample, thus limiting generalizability. Nonetheless, this first exploration on a micro level of the patterns between therapist coaching and parental skill acquisition remain important.

## Conclusion

This study is the first to explore the bidirectional nature of interactions between therapists and parents during in vivo coaching in PCIT. Findings suggest that parents immediately increase their use of positive parenting skills when therapists use responsive coaching to reinforce a previous verbalization. In other words, positive reinforcement of a positive parenting skill during live coaching promotes the use of that specific skill. This study, which included a sample of Dutch therapists and families, adds to the accumulating evidence that both responsive and directive therapist coaching lead to parents’ acquisition of child-centered skills. For the first time, it is possible to see on a micro level that parents are immediately influenced by responsive coaching. Additionally, the parents’ use of child-centered skills was also positively influenced by directive coaching techniques. Nonetheless, we must be cautious with directive coaching due to previous studies relating early use of directive coaching to attrition (Heymann et al., 2022). This pleads for therapists to responsibly balance between responsive and directive coaching strategies throughout sessions. We reveal that therapist coaching techniques are complex and warrant further exploration. Therefore, future research should continue to explore the bidirectional interactions between therapist and parent verbalization to gain more understanding of coaching and its effects. This study is a first step toward revealing, at a micro level, the power of coaching techniques. Ultimately, better understanding of therapist-parent interactions may help to promote effective therapist coaching and positive outcomes for families.

## References

[bibr1-01454455251319731] Abrahamse M. E. Junger M. Leijten P. H. Lindeboom R. Boer F. Lindauer R. J. (2015). Psychometric properties of the Dutch Eyberg Child Behavior Inventory (ECBI) in a community sample and a multi-ethnic clinical sample. Journal of Psychopathology and Behavioral Assessment, 37, 679–691. 26640320 10.1007/s10862-015-9482-1 PMC4661191

[bibr2-01454455251319731] Abrahamse M. E. Junger M. van Wouwe M. A. Boer F. Lindauer R. J. (2016). Treating child disruptive behavior in high-risk families: A comparative effectiveness trial from a community-based implementation. Journal of child and family studies, 25(5), 1605–1622. 27110086 10.1007/s10826-015-0322-4 PMC4824803

[bibr3-01454455251319731] Abrahamse M. E. Niec L. N. Solomon D. T. Junger M. Lindauer R. J. (2019). Psychometric properties of the dyadic parent-child interaction coding system in The Netherlands. Child & Family Behavior Therapy, 41(3), 141–158.

[bibr4-01454455251319731] Abrahamse M. E. Tsang V. M. Lindauer R. J. (2021). Home-based parent–child interaction therapy to prevent child maltreatment: A randomized controlled trial. International Journal of Environmental Research and Public Health, 18(16), 8244. 34444004 10.3390/ijerph18168244 PMC8394039

[bibr5-01454455251319731] Barnett M. L. Davis E. M. Schoonover C. E. Niec L. N. (2018). Therapist–parent interactions in PCIT: The importance of coach coding. In Niec L. (Ed.), Handbook of parent-child interaction therapy (pp. 303–317). Springer.

[bibr6-01454455251319731] Barnett M. L. Niec L. N. Acevedo-Polakovich I. D. (2014). Assessing the key to effective coaching in parent–child interaction therapy: The therapist-parent interaction coding system. Journal of Psychopathology and Behavioral Assessment, 36(2), 211–223. 24839350 10.1007/s10862-013-9396-8 PMC4019441

[bibr7-01454455251319731] Barnett M. L. Niec L. N. Peer S. O. Jent J. F. Weinstein A. Gisbert P. Simpson G. (2017). Successful therapist–parent coaching: How in vivo feedback relates to parent engagement in parent–child interaction therapy. Journal of Clinical Child & Adolescent Psychology, 46(6), 895–902. 26467101 10.1080/15374416.2015.1063428

[bibr8-01454455251319731] Eyberg S. Funderburk B. (2011). Parent-child interaction therapy protocol. PCIT International.

[bibr9-01454455251319731] Eyberg S. M. Nelson M. Duke M. Boggs S. R. (2004). Manual for the dyadic parent-child interaction coding system third edition. Unpublished Rating Manual.

[bibr10-01454455251319731] Eyberg S. Pincus D. (1999). ECBI & SESBI-R: Eyberg child behavior inventory and Sutter-Eyberg student behavior inventory-revised: Professional manual. Psychological Assessment Resources.

[bibr11-01454455251319731] Greco L. A. Sorrell J. T. McNeil C. B. (2001). Understanding manual-based behavior therapy: Some theoretical foundations of parent-child interaction therapy. Child & Family Behavior Therapy, 23(4), 21–36.

[bibr12-01454455251319731] Green Rosas Y. McCabe K. M. Zerr A. Yeh M. Gese K. Barnett M. L . (2022). Examining English-and Spanish-speaking therapist behaviors in parent–child interaction therapy. International Journal of Environmental Research and Public Health, 19(8), 4474. 35457342 10.3390/ijerph19084474 PMC9031310

[bibr13-01454455251319731] Hakman M. Chaffin M. Funderburk B. Silovsky J. F. (2009). Change trajectories for parent-child interaction sequences during parent-child interaction therapy for child physical abuse. Child Abuse & Neglect, 33(7), 461–470. 19581001 10.1016/j.chiabu.2008.08.003

[bibr14-01454455251319731] Heymann P. Heflin B. H. Bagner D. M. (2022). Effect of therapist coaching statements on parenting skills in a brief parenting intervention for infants. Behavior Modification, 46(4), 691–705. 33448233 10.1177/0145445520988140 PMC8280235

[bibr15-01454455251319731] Kaminski J. W. Valle L. A. Filene J. H. Boyle C. L. (2008). A meta-analytic review of components associated with parent training program effectiveness. Journal of Abnormal Child Psychology, 36(4), 567–589. 18205039 10.1007/s10802-007-9201-9

[bibr16-01454455251319731] Leijten P. Gardner F. Melendez-Torres G. Van Aar J. Hutchings J. Schulz S. Knerr W. Overbeek G. (2019). Meta-analyses: Key parenting program components for disruptive child behavior. Journal of the American Academy of Child & Adolescent Psychiatry, 58(2), 180–190. 30738545 10.1016/j.jaac.2018.07.900

[bibr17-01454455251319731] Leitão S. M. Seabra-Santos M. J. Gaspar M. F. (2021). Therapist factors matter: A systematic review of parent interventions directed at children’s behavior problems. Family Process, 60(1), 84–101. 32413195 10.1111/famp.12550

[bibr18-01454455251319731] Meyers L. S. Gamst G. C. Guarino A. (2013). Performing data analysis using IBM SPSS. John Wiley & Sons.

[bibr19-01454455251319731] Niec L. N. Barnett M. L. Peer S. Schoonover C. Boog K. (2016). Comprehensive manual for the therapist-parent interaction coding system. Unpublished manual, Central Michigan University.

[bibr20-01454455251319731] Noldus L. (1991). The observer: A software system for collection and analysis of observational data. Behavior Research Methods, Instruments, & Computers, 23(3), 415–429. 10.3758/bf03195516 14587547

[bibr21-01454455251319731] Shanley J. R. Niec L. N. (2010). Coaching parents to change: The impact of in vivo feedback on parents’ acquisition of skills. Journal of Clinical Child & Adolescent Psychology, 39(2), 282–287. 20390820 10.1080/15374410903532627

